# Total and individual antioxidant intake and risk of epithelial ovarian cancer

**DOI:** 10.1186/1471-2407-12-211

**Published:** 2012-06-01

**Authors:** Dina Gifkins, Sara H Olson, Lisa Paddock, Melony King, Kitaw Demissie, Shou-En Lu, Ah-Ng Tony Kong, Lorna Rodriguez-Rodriguez, Elisa V Bandera

**Affiliations:** 1 The Cancer Institute of New Jersey, Robert Wood Johnson Medical School, 195 Little Albany St., New Brunswick, NJ, 08903, USA; 2 School of Public Health, University of Medicine and Dentistry of New Jersey, Piscataway, NJ, USA; 3 Department of Epidemiology and Biostatistics, Memorial Sloan-Kettering Cancer Center, New York, NY, USA; 4 New Jersey Department of Health and Senior Services, Trenton, NJ, USA; 5 Rutgers University, Department of Pharmaceutics, Ernest Mario School of Pharmacy, Piscataway, NJ, USA

**Keywords:** Ovarian neoplasms, Antioxidants, Total antioxidant capacity, Vitamin C, Vitamin E, Beta-carotene, Selenium, Lutein, Lycopene, Diet

## Abstract

**Background:**

Limiting oxidative stress to the ovarian epithelium has been proposed as a first-line defense against ovarian cancer. Although evidence for an association between individual dietary antioxidant intake and ovarian cancer risk is conflicting, the combined evidence suggests a modest inverse association. Our study aimed to evaluate the association between total antioxidant capacity (TAC) and individual antioxidant intakes (vitamin C, vitamin E, beta-carotene, selenium, lutein, and lycopene) and ovarian cancer risk.

**Methods:**

We conducted a population-based case–control study in New Jersey. Cases were women ages 21 years and older with newly diagnosed epithelial ovarian cancer who resided in six counties of New Jersey. Controls were women in the same age range who resided in the same geographic area. A total of 205 ovarian cancer cases and 390 controls were included. Dietary intake was ascertained using the Block food frequency questionnaire (FFQ), and TAC indices were constructed by linking FFQ-derived estimates to two standardized antioxidant capacity databases, the USDA Oxygen Radical Absorbance Capacity (ORAC) Database, and the University of Olso’s Antioxidant Food Database. Multivariate logistic regression models were used to calculate odds ratios and 95 % confidence intervals while controlling for major ovarian cancer risk factors.

**Results:**

We found a strong inverse association with selenium from food sources (OR: 0.41; 95 % CI: 0.20-0.85, for the highest vs. lowest tertile of dietary selenium intake). However, there was little evidence of an association with dietary TAC or the others individual antioxidants. In contrast, compared to non-users, supplement users had significant increased risk for all micronutrients, but no statistically significant increased risk was observed for combined intake from foods and supplements of any of these antioxidants.

**Conclusions:**

This study found an inverse association between selenium consumption from food sources and ovarian cancer risk, while there was little evidence of an association with TAC or any of the other individual antioxidants. Additional research is needed to confirm these findings.

## Introduction

Ovarian cancer is associated with the highest mortality among all gynecologic cancers [1]. Ovarian cancer is commonly referred to as a “silent killer”, as symptoms are often non-specific and women are therefore diagnosed in late stages of disease. Approximately 21,990 women were diagnosed in 2011, and 15,460 women died from ovarian cancer [1].

In ovarian cancer, more than 90 % of cancers originate from the surface epithelial cells [2]. The etiologic theory of incessant ovulation was proposed in the 1970’s by Fathalla [3], which suggests that repeated ovulations are responsible for transformation of the ovarian epithelium. Additional studies have shown that ovarian surface epithelial cells surrounding follicular rupture are exposed to mutagens, including both inflammatory mediators and oxidants, which are produced during the periovulatory period [4,5]. During the process of ovulation and luteinization, reactive oxidants are produced in excess. Additionally, processes which occur during periovulatory remodeling cause ovarian surface epithelial cells to suffer genomic damages leading to apoptosis, and surrounding cells are exposed to excessive reactive oxidants. Animal and human experimental studies have confirmed that surface ovarian epithelial cells contain elevated levels of 8-oxoguanine during ovulation, which is one of the most important mutagenic lesions in DNA [6,7]. It has therefore been suggested that limiting oxidative stress to the ovarian epithelium could be considered a first-line defense against ovarian cancer [6].

Although evidence for an association between individual dietary antioxidant intake and ovarian cancer risk is conflicting [8], the combined role of these micronutrients in the prevention of ovarian cancer has not been elucidated. As both individual contributions from micronutrients and combined additive or synergistic effects may alter risk, understanding the combined effect of antioxidant micronutrients on the risk of ovarian cancer may also help to develop a better understanding of their role in disease prevention.

We therefore sought to investigate the association between total antioxidant capacity (TAC) intake and ovarian cancer risk using data from standardized antioxidant databases in a population-based case–control study conducted in New Jersey [9,10]. As few studies have investigated the role of antioxidant supplement intake and ovarian cancer risk, and evidence on individual dietary antioxidants is inconclusive, we also investigated the association between individual antioxidant micronutrients from food and supplements with ovarian cancer risk.

## Methods

The *NJ Ovarian Cancer Study* has been described in detail elsewhere [9,10]. It was designed using identical study instruments and a shared control group with the EDGE Study (Estrogen, Diet, Genetics, and Endometrial Cancer) [11,12], and was conducted in a collaboration between the Cancer Institute of New Jersey (CINJ), Memorial Sloan-Kettering Cancer Center (MSKCC), and the New Jersey State Cancer Registry (NJSCR), based at the New Jersey Department of Health and Senior Services (NJDHSS).

In brief, cases were women with newly diagnosed histologically confirmed invasive epithelial ovarian cancer identified between January 2004 and May 2008. Ovarian cancer cases aged 21 years or older who spoke English or Spanish and resided in six counties of New Jersey (Bergen, Essex, Hudson, Middlesex, Morris, or Union) were eligible to participate. Case ascertainment was conducted by the NJSCR using rapid case ascertainment, supplemented with review of NJSCR data to identify cases diagnosed out of the area. During the study period, 493 women were identified. Of those, 61 passed away before they could be interviewed, for 9 cases their physicians advised that we should not contact them, and an additional 119 cases were not able to participate for various reasons (e.g., found to be ineligible, unable to reach, communication barriers, comorbidities). Of the remaining women, 233 (47 %) ovarian cancer cases completed the interview.

Controls were women 21 years and older who resided in the same six counties as the cases (Bergen, Essex, Hudson, Middlesex, Morris, or Union) and spoke English or Spanish. Women who had a hysterectomy and/or a bilateral oophorectomy were not eligible to participate. To identify controls aged < 65 years, random digit dialing was conducted. Of the 355 eligible women identified, 175 (49 %) completed the interview. To identify women > 65 years of age, random selection was conducted from lists purchased from the Centers for Medicare and Medicaid. Of the 316 women who were contacted, 68 (22 %) completed the interview. However, the eligibility of 40 % was unknown. We conducted area sampling to identify additional controls older than 65 years, starting in 2003. Thirty consecutive households in randomly chosen neighborhoods were contacted by mail and by home visits. To better match the age distribution of the cases women aged 55 years and older were later included. Overall, 467 (40 %) controls completed the interview.

Informed consent was provided by all women participating in the studies, and IRB approval was obtained from the CINJ, MSKCC, and the NJDHSS.

### Data collection

A questionnaire was administered to participants to collect data on potential and established ovarian cancer risk factors. Information on demographic characteristics, residential history, pregnancy history, occupation, oral contraceptive use and other birth control methods, menstrual history and menopausal status, personal and family history of cancer and other illnesses, height and weight, physical activity, and exposure to other potential risk factors was included.

To assess dietary intake, participants were asked to complete the Block Food Frequency Questionnaire (FFQ), version 98.2 (NutritionQuest, Berkeley, CA). Patients reported their usual dietary intake in the six months prior to their date of entry into the study for controls and their diagnosis date for cases. A total of 205 ovarian cancer cases and 398 controls completed the FFQ. Eight controls were further excluded as they had both ovaries removed and were therefore ineligible, leaving 390 controls available for analysis.

NutritionQuest provided nutrient calculations for individual antioxidants using the USDA Nutrient Database for Standard Reference. We derived TAC indices by linking FFQ-derived food consumption to two standardized antioxidant capacity databases, the USDA Oxygen Radical Absorbance Capacity (ORAC) Database (http://www.ars.usda.gov/sp2userfiles/place/12354500/data/orac/orac07.pdf), and the University of Olso’s Antioxidant Food Database [13].

Total daily intake of food items known to be high in antioxidant content from the FFQ, including over 50 food items, such as fruits, vegetables, juices, grains, teas, chocolate and wine was computed first. Daily consumption was derived based on frequency and portion size, and food values were converted from their unit listed in the FFQ to grams per day, using values from the USDA Nutrient Database for Standard References (http://www.nal.usda.gov).

The ORAC database, developed by the USDA Agricultural Research Service, was utilized to develop individual indices for TAC: total antioxidant capacity (T-ORAC), hydrophilic antioxidant capacity (H-ORAC), lipophilic antioxidant capacity (L-ORAC), and total phenolics (TP). Additionally, antioxidant values were derived using another database, the Antioxidant Food (FRAP) database, which is based on the electron transfer FRAP assay to extract antioxidant values. Both databases were used to assess the comparability of results using the two assays.

Antioxidant values were attributed to each food item based on the data in the antioxidant databases. The total *u*mol of Trolox Equivalents per 100 grams per day for each food item and total mg gallic acid equivalents per 100 grams per day for each food item were calculated, and the total of all items were summed to develop each of the indices. Separate antioxidant indices were developed for total H-ORAC, L-ORAC, T-ORAC, and TP, from the ORAC database, and a TAC index was developed from the Antioxidant Food (FRAP) Database. As the ORAC database does not include values for antioxidant supplements, to compare the results of the TAC index from the ORAC database to that of the FRAP database, a TAC index without supplements based on the FRAP index was also developed.

### Data analysis

The relationship between ovarian cancer and the TAC indices and individual antioxidants (vitamin C, vitamin E, beta-carotene, lycopene, lutein, and selenium, from food sources, supplements, and food and supplements combined) was assessed by evaluating the indices as categorical variables. Tertiles of each TAC index were created based on the distribution of each index among controls. Age-adjusted ANCOVA and cross-tabulations were conducted to assess any association between the TAC indices and case–control status. Multiple unconditional logistic regression models were developed to estimate odds ratios and 95 % confidence intervals while controlling for potential confounders. Potential covariates included age, body mass index (BMI), education (high school or less, college, graduate school), race, age at menarche (continuous), menopausal status, parity (0–1, 2, >3), oral contraceptive use (ever, never), hormone replacement therapy (HRT) use (never, used unopposed estrogen only, used combined therapy), tubal ligation, total energy intake (continuous), smoking status (never, current, former), alcohol consumption (continuous), and physical activity (continuous total metabolic equivalents). Tests for trend were derived by assigning a median value to each tertile of the antioxidant variables. All analyses were conducted using SAS software version 9.2.

## Results

Demographic and patient characteristics are presented in Table 1. The majority of participants were white. As expected, odds ratios were below one for oral contraceptive use, higher parity, and a prior tubal ligation, and above one for having a first degree relative with ovarian cancer.

**Table 1:** Selected characteristics of women participating in The NJ Ovarian Cancer Study

	**Cases (*n*** **= 205) n (%)**		**Controls (*n*** **= 391) n (%)**		OR (95 % CI)*
**Education**					
High school or less	61	(29.8)	133	(34.0)	1.00 (Ref)
College	93	(45.4)	159	(40.7)	0.90 (0.59-1.38)
Graduate school	51	(24.9)	99	(25.3)	0.76 (0.47-1.24)
**Race/ethnicity**					
White	179	(87.3)	344	(88.4)	1.00 (Ref)
Black	9	(4.4)	17	(4.4)	1.02 (0.42-2.44)
Other	8	(3.9)	17	(4.4)	0.82 (0.33-1.99)
Hispanic (any race)	9	(4.4)	11	(2.8)	1.13 (0.44-2.92)
**BMI**					
Underweight (<18.5)	1	(0.5)	1	(0.3)	1.02 (0.06-17.31)
Normal (18.5-25)	90	(43.9)	180	(46.4)	1.00 (Ref)
Overweight (25–29.9)	54	(26.3)	122	(31.4)	1.07 (0.69-1.65)
Obese (30–34.9)	36	(17.6)	59	(15.2)	1.39 (0.83-2.32)
Very obese (>35)	24	(11.7)	26	(6.7)	1.54 (0.81-2.89)
**Parity**					
0 – 1	97	(47.3)	92	(23.5)	1.00 (Ref)
2	60	(29.3)	137	(35.0)	0.45 (0.29-0.69)
>3	48	(23.4)	162	(41.4)	0.42 (0.26-0.66)
**Smoking status**					
Never	108	(52.7)	204	(52.2)	1.00 (Ref)
Past	78	(38.1)	149	(38.1)	1.12 (0.76-1.64)
Current	19	(9.3)	38	(9.7)	0.87 (0.46-1.62)
**Oral contraceptive use**					
Never	85	(41.5)	193	(49.4)	1.00 (Ref)
Ever	120	(58.5)	198	(50.6)	0.88 (0.61-1.28)
**Use of HRT**					
Never	159	(77.6)	285	(72.9)	1.00 (Ref)
Unopposed E only	22	(10.7)	34	(8.7)	1.56 (0.86-2.84)
Any combined HRT	24	(11.7)	72	(18.4)	0.63 (0.38-1.06)
**Age at menarche**					
>13	41	(20.1)	99	(25.4)	0.81 (0.51-1.27)
12-13	117	(57.4)	200	(51.3)	1.00 (Ref)
<11	46	(22.6)	91	(23.3)	0.75 (0.48-1.17)
**Menopause status**					
Premenopausal	71	(34.6)	49	(12.5)	1.51 (0.85-2.69)
Postmenopausal					
Age at menopause					
<40	5	(2.4)	14	(3.6)	0.77 (0.26-2.31)
41-54	86	(42.0)	239	(61.3)	1.00 (Ref)
>55	12	(5.9)	37	(9.5)	0.99 (0.48-2.01)
Unknown	31	(15.1)	52	(13.3)	1.52 (0.91-2.56)
**Tubal Ligation**					
No	175	(85.4)	315	(80.6)	1.00 (Ref)
Yes	30	(14.6)	76	(19.4)	0.59 (0.36-0.94)
**First relative with ovarian cancer**					
No	195	(95.1)	377	(96.4)	1.00 (Ref)
Yes	10	(4.9)	14	(3.6)	1.32 (0.55-3.17)

^*^Adjusted for age.

Tables 2 and 3 show the age-adjusted mean intake for each TAC index and individual antioxidant micronutrients among cases and controls. Cases tended to have a slightly lower intake of total antioxidants. However, no significant differences were observed between cases and controls for any of the TAC indices or individual micronutrients.

**Table 2:** Age-adjusted mean total antioxidant capacity intake based on antioxidant indices in cases and controls

	**Mean (SE)**		
	**Cases**	**Controls**	***p*value**
H-ORAC, *u*molTE/100 g	13,000 (504)	13,488 (361)	0.44
L-ORAC, *u*molTE/100 g	394 (20)	437 (14)	0.08
T-ORAC, *u*molTE/100 g	13,275 (514)	13,809 (368)	0.41
TP, mgGAE/100 g	1776 (71)	1821 (50)	0.61
FRAP, *u*molTE/100 g	5,361 (214)	5,446 (153)	0.75
FRAP, with supplements, *u*molTE/100 g	13,051 (807)	12,716 (577)	0.74

H-ORAC: Hydrophilic oxygen radical absorbance capacity; L-ORAC: Lipophilic oxygen radical absorbance capacity; T-ORAC: Total oxygen radical absorbance capacity; TP: Total phenolics; TE: Trolox equivalents; GAE: Gallic acid equivalents; SE: Standard error.

**Table 3:** Age-adjusted mean antioxidant micronutrient intake from foods and supplements

	**Mean (SE)**		
	**Cases**	**Controls**	***p*value (*t*test)**
Vitamin C, mg/1,000 kcal			
Food	117.5 (5.0)	119.4 (3.6)	0.76
Supplements	309.4 (31.9)	281.9 (22.8)	0.49
Total intake	426.9 (32.8)	401.3 (23.4)	0.54
Vitamin E, aTE/1,000 kcal			
Food	10.0 (0.4)	10.3 (0.3)	0.46
Supplements	83.3 (10.8)	105.8 (7.8)	0.10
Total intake	93.3 (10.9)	116.1 (7.8)	0.09
Beta-carotene, mcg/1,000 kcal			
Food	3507.6 (188.8)	3481.6 (135.1)	0.91
Supplements	2531.9 (424.4)	2294.4 (303.7)	0.66
Total intake	6039.5 (477.5)	5776.0 (341.7)	0.66
Selenium, mcg/1,000 kcal			
Food sources	74.9 (2.6)	78.2 (1.9)	0.32
Supplements	23.2 (3.3)	22.4 (2.4)	0.85
Total intake	98.1 (4.5)	100.5 (3.2)	0.65
Lutein (food), mcg/1,000 kcal	1750.3 (108.9)	1859.2 (77.9)	0.42
Lycopene (food), mcg/1,000 kcal	5454.1 (394.3)	5045.2 (282.2)	0.41

SE: Standard error; aTE: alpha tocopherol equivalents.

After adjusting for major risk factors, there was little evidence of an association with any of the TAC indices, as shown in Table 4. No association was observed for consumption of vitamin C, vitamin E, or beta-carotene from food sources, as shown in Table 5. However, intake of selenium from food sources was associated with a strong, significant inverse association with ovarian cancer risk (OR: 0.41; 95 % CI: 0.20-0.85 for the highest tertile compared to the lowest). In contrast, compared to non-users, ovarian cancer risk was elevated for supplement users of all individual antioxidants examined, with ORs (95 % CI) of 1.63 (1.01-2.62) for vitamin C, 1.63 (1.02-2.63) for vitamin E, 1.69 (1.08-2.66) for beta-carotene, and 1.64 (1.05-2.56) for selenium supplement users. No association was observed when the amount of antioxidant supplement intake was combined with dietary intake. There was a suggestion of increased risk for dietary lycopene (OR: 1.54; 95%CI: 0.90-2.62) and lutein (OR: 1.48; 95%CI: 0.82-2.68), but it was not statistically significant.

**Table 4:** Total antioxidant capacity intake based on antioxidant indices and ovarian cancer risk

	**Cases (*n*)**	**Controls (*n*)**	**OR1**	**95 % CI**	**OR2**	**95 % CI**
**ORAC Database**						

H-ORAC (*u*molTE/100 g)						
1 (<9,505)	75	128	1.00		1.00	
2 (9,505-15,552)	65	132	0.93	0.58-1.49	0.93	0.55-1.56
3 (>15,553)	65	127	1.01	0.64-1.77	1.19	0.69-2.01
*p* for trend				0.68		0.65
L-ORAC (*u*molTE/100 g)						
1 (<275.9)	74	127	1.00		1.00	
2 (275.9-470.4)	73	132	0.87	0.54-1.41	0.83	0.48-1.41
3 (>470.5)	58	128	0.84	0.49-1.44	0.91	0.50-1.64
*p* for trend				0.62		0.48
T-ORAC (*u*molTE/100 g)						
1 (<9,760)	74	127	1.00		1.00	
2 (9,760-16,040)	70	133	1.01	0.63-1.61	1.03	0.61-1.72
3 (>16,041)	61	127	0.98	0.58-1.64	1.09	0.62-1.94
*p* for trend				0.95		0.96
Total phenolics (mgGAE/100 g)						
1 (<1,315)	85	131	1.00		1.00	
2 (1,315-2,180)	64	134	0.88	0.55-1.40	1.13	0.68-1.88
3 (>2,181)	56	130	0.84	0.50-1.41	0.88	0.49-1.59
*p* for trend				0.66		0.54
**FRAP** (*u*molTE/100 g)						
1 (<3,951)	52	127	1.00		1.00	
2 (3,951-6,482)	82	133	0.81	0.51-1.31	0.92	0.55-1.53
3 (>6,483)	71	127	1.00	0.61-1.65	1.07	0.62-1.86
*p* for trend				0.36		0.66
FRAP with supplements (*u*molTE/100 g)						
1 (<6,261)	52	127	1.00		1.00	
2 (6,261-14,110)	81	133	1.20	0.79-2.06	1.18	0.65-2.14
3 (>14,111)	72	127	1.15	0.63-2.08	1.27	0.66-2.43
*p* for trend				0.52		0.65

ORAC: Oxygen radical absorbance capacity; H-ORAC: Hydrophilic oxygen radical absorbance capacity; L-ORAC: Lipophilic oxygen radical absorbance capacity; T-ORAC: Total oxygen radical absorbance capacity; TE: Trolox equivalents;GAE: gallic acid equivalents; OR: Odds Ratio; CI: Confidence interval.

OR1: adjusted for age (continuous), education, race, age at menarche (continuous), menopausal status and age at menopause for postmenopausal women, parity, OC use, HRT use, BMI (continuous), tubal ligation, antioxidant supplement intake, and total calories; OR2: further adjusted for physical activity (METs), and smoking status.

**Table 5:** Antioxidant micronutrients from food and supplements and ovarian cancer risk

	**Cases (n)**	**Controls(n)**	**OR1**	**95 % CI**	**OR2**	**95 % CI**
**Vitamin C**						
Food (mg)						
1 (<82.3)	75	127	1.00		1.00	
2 (82.3-141.7)	72	132	0.93	0.58-1.48	1.08	0.65-1.79
3 (>141.8)	58	128	0.99	0.59-1.67	1.29	0.72-2.29
*p* for trend				0.75		0.89
Supplements						
No	56	111	1.00		1.00	
Yes	149	276	1.34	0.87-2.06	1.63	1.01-2.62
Combined food and supplements (mg)						
1 (<147.5)	74	128	1.00		1.00	
2 (147.5-458.6)	72	132	1.09	0.69-1.74	1.38	0.83-2.31
3 (>458.7)	59	127	1.06	0.66-1.71	1.42	0.84-2.40
*p* for trend				0.72		0.28
**Vitamin E**						
Food (aTE)						
1 (<7.4)	69	128	1.00		1.00	
2 (7.4-11.5)	64	132	0.96	0.56-1.65	0.94	0.53-1.67
3 (>11.6)	72	127	1.04	0.55-1.98	0.89	0.45-1.77
*p* for trend				0.84		0.89
Supplements						
No	58	114	1.00		1.00	
Yes	147	273	1.35	0.88-2.07	1.63	1.02-2.63
Combined food and supplements (aTE)						
1 (<21.7)	69	128	1.00		1.00	
2 (21.7-114.8)	88	131	1.53	0.98-2.41	1.81	1.10-1.04
3 (>114.9)	48	128	0.87	0.52-1.42	1.03	0.59-1.78
*p* for trend				0.29		0.10
**Beta-carotene**						
Food (mcg)						
1 (<2,070)	67	128	1.00		1.00	
2 (2,070-3,675)	66	132	1.14	0.70-1.86	1.05	0.61-1.78
3 (>3,676)	72	127	1.35	0.81-2.25	1.45	0.83-2.52
*p* for trend				0.61		0.91
Supplements						
No	67	138	1.00		1.00	
Yes	138	249	1.40	0.93-2.11	1.69	1.08-2.66
Combined food and supplements (mcg)						
1 (<3,040)	79	128	1.00		1.00	
2 (3,040-5,075)	57	132	0.82	0.51-1.33	0.87	0.52-1.47
3 (>5,076)	69	127	1.09	0.67-1.79	1.38	0.80-2.35
*p* for trend				0.33		0.41
**Selenium**						
Food (mcg)						
1 (<59.5)	84	128	1.00		1.00	
2 (59.5-87.7)	58	132	0.48	0.28-0.81	0.55	0.31-0.98
3 (>87.8)	63	127	0.40	0.21-0.78	0.41	0.20-0.85
*p* for trend				0.01		0.10
Supplements						
No	69	148	1.00		1.00	
Yes	136	239	1.44	0.95-2.15	1.64	1.05-2.56
Combined food and supplements (mcg)						
1 (<72.4)	79	128	1.00		1.00	
2 (72.4-103.6)	58	132	0.69	0.42-1.16	0.73	0.42-1.27
3 (>103.7)	68	127	0.73	0.40-1.30	0.75	0.39-1.43
*p* for trend				0.20		0.30
**Lutein**						
Food (mcg)						
1 (<1,019)	65	128	1.00		1.00	
2 (1,019-1,882)	65	131	1.21	0.74-1.97	1.28	0.75-2.19
3 (>1,882)	75	128	1.46	0.89-2.39	1.54	0.90-2.62
*p* for trend				0.63		0.51
**Lycopene**						
Food (mcg)						
1 (<2,504)	51	127	1.00		1.00	
2 (2,504-5,464)	83	133	1.68	1.01-2.79	1.43	0.83-2.47
3 (>5,465)	71	127	1.58	0.91-2.74	1.48	0.82-2.68
*p* for trend				0.05		0.26

OR1: adjusted for age (continuous), education, race, age at menarche (continuous), menopausal status and age at menopause for postmenopausal women, parity, OC use, HRT use, BMI (continuous), tubal ligation, and total calories; OR2: further adjusted for physical activity (METs) and smoking status.

## Discussion

In this study we found little evidence that the dietary TAC had an impact on ovarian cancer risk, based on antioxidant content values from the USDA ORAC database and the FRAP-based Antioxidant Food Database. In line with these findings, most individual micronutrients evaluated were also not found to be associated with ovarian cancer, with the exception of selenium. We found a statistically significant 60 % decreased risk of ovarian cancer for women in the highest tertile of selenium intake from food sources compared to the lowest. In contrast, we observed significant increased risk for users of all types of antioxidant supplements examined, compared to non-users. However, no increased risks were observed for the total amount of antioxidant intake when supplement amounts were combined with dietary intake.

Similar to our results, most studies investigating dietary vitamin C and ovarian cancer risk report no association [14-23]. However, some studies have found significant decreased risks [24-28]. The only two cohort studies evaluating vitamin C reported no association, including an analysis of the Nurse’s Health Study, which identified 301 ovarian cancer cases during a follow-up of 16 years [23]. Additionally, a meta-analysis of case-controls studies found no association, reporting a pooled OR of 0.98 (95 % CI: 0.95-1.01) for additional daily intake of 30 mg vitamin C [8]. Only one other study has investigated supplement use, and in contrast to our results found a significantly decreased risk of almost 50 %, and also reported a decreased risk for supplements combined with dietary intake [18].

We also found no association with vitamin E intake. Similarly, of all previously conducted studies evaluating vitamin E and ovarian cancer risk [14-18,21-24,26,29-31], most found no association. Only a few case–control studies reported decreased risks [17,24,26,31]. In contrast to our findings, the only two studies investigating supplement use reported strong inverse associations [18,30].

Consistent with our findings, several cohort studies [14,15,22,23] and some case–control studies [16,18,32] found no association with beta-carotene from food sources. Also, a meta-analysis of case–control studies reported no association (pooled OR: 0.98; 95 % CI: 0.96-0.99) for additional daily intake of 500mcg beta-carotene [8]. However, an inverse association with beta-carotene has been reported in other studies [17,26,27,29,31], and non-significant decreased risks have also been reported [21,33]. To our knowledge, our study is the first to investigate beta-carotene supplement use and ovarian cancer risk.

In support of our findings, a nested case–control study investigating the serum selenium status of ovarian cancer patients compared to healthy controls found that increasing serum selenium was associated with a decreased risk of ovarian cancer (OR: 0.23; 95%CI: 0.1-0.9 for highest tertile compared to lowest; p for trend = 0.02) [34]. Das and Ma (1986) reported similar findings, with a reverse correlation between serum selenium concentration and ovarian cancer incidence[35]. Additionally, Sieja (1998) found ovarian cancer patients had significantly lower serum selenium concentrations compared to controls (p < 0.05), approaching critical levels, particularly during chemotherapy treatment[36]. However, other studies investigating selenium intake from dietary and supplement sources [14,18,21], including an analysis of the Women’s Health Initiative [14], found no association with ovarian cancer risk. Tung et al. [21] assessed selenium intake among a multiethnic group of patients from Hawaii and Los Angeles and Fleischauer et al. [18] had a smaller number of cases (n = 169), and used both community and hospital-based controls. Differences in the study populations may partially explain the conflicting results.

Numerous studies have indicated that selenium may have anticancer properties. Human studies have shown a decrease in the incidence of prostate, lung, and colorectal cancers [37,38]. Additionally, animal studies have shown that, at high doses, selenium compounds reduce tumor yields, inhibit cell growth, and stimulate programmed cell death in cultures. In ovarian cancer cells, it has been demonstrated that selenium compounds inhibit the synthesis of nucleic acids (Rzaeva, 1985). Selenoprotein deficiency has also been found to be present in certain types of cancer. The proposed mechanism by which selenium may have a protective effect on cancer is mainly through its antioxidant properties. Selenium provides antioxidant protection against the effect of reactive oxygen species on cancer initiation and promotion [39]. Geographic variation in selenium concentrations exists, however no study has investigated whether ovarian cancer risk differs in areas with varying selenium concentrations.

Our conflicting findings of a protective effect associated with dietary selenium intake versus an increased risk associated with supplement intake are unclear. Of note, the increased risk observed with selenium supplements only reached statistical significance after further adjustment for physical activity and smoking status. It is possible that related unmeasured confounders may be influencing the association. Studies of supplement intake and relation with cancer risk have been conflicting. A recent systematic review concludes that evidence does not support selenium supplementation in the prevention of cancer [40]. Other reports have suggested that certain supplements may increase the risk of cancer, as has been recently observed with vitamin E supplementation and prostate cancer risk [41].

Only one other study has developed a total antioxidant score in ovarian cancer patients, using data from the Teacher’s Health Study in California [15]. However, this study only included antioxidant values from fruits and vegetables, identified from variable literature sources, and did not assess supplement use. Similar to our results, no association was observed.

This study may be subject to the limitations of case–control studies, such as recall bias and selection bias. Particularly with our finding of increased risk with supplements, it is possible that cases may have reported use differently than controls. We also had a low participation rate. Potential non-participation bias was assessed by comparing characteristics of women who participated in this study to cases who did not in the New Jersey State Cancer Registry during the study time period. Cases that consented tended to be younger; however the racial/ethnic distribution and distributions by histology, stage and grade were similar. We did not have information on women who did not end up participating as controls. However, the distribution of risk factors in this study is similar to that reported in other studies, which gives us reassurance in the validity of our data.

In conclusion, this study found a strong inverse association of selenium from food sources and ovarian cancer risk, while selenium supplement intake was associated with increased risk. However, we did not find any significant association between TAC intake and ovarian cancer risk. Supplement use in this study was found to be associated with increased ovarian cancer risk for all supplements studied. However, combined amounts of antioxidants from diet and supplement sources were not associated with increased risks. As several reports have recently raised concerns about the safety of vitamin supplements in recent years, including the 2007 WCRF/AICR Report which warned cancer patients and survivors against taking certain supplements [8], these findings warrant further investigation to better understand the role of selenium from foods and supplements on ovarian cancer risk.

## Competing interests

The authors declare that they have no competing interests.

## Authors’ contributions

DG wrote the first draft of the manuscript. EVB conceptualized the study and provided overall supervision for conducting the study. DG, EVB, MK conducted data analyses. LEP implemented case ascertainment. Additional expertise was provided by KD, SHO (cancer epidemiology), LRR (gynecologic oncology), S-EL (biostatistics), A-NTK (antioxidants, carcinogenesis). All authors revised the article critically for important intellectual content and approved the final version of the manuscript.

## Pre-publication history

The pre-publication history for this paper can be accessed here:

http://www.biomedcentral.com/1471-2407/12/211/prepub
